# Efficacy and safety of artemisinin-based antimalarial in the treatment of uncomplicated malaria in children in southern Tanzania

**DOI:** 10.1186/1475-2875-6-146

**Published:** 2007-11-11

**Authors:** Abdunoor M Kabanywanyi, Alex Mwita, Deborah Sumari, Renata Mandike, Kefas Mugittu, Salim Abdulla

**Affiliations:** 1 Ifakara Health Research and Development Centre, Tanzania; 2 National Malaria Control Programme, Ministry of Health and Social Welfare, United Republic of Tanzania; 3 Faculty of Science Department of Molecular biology and Biotechnology, University College of Dar es Salaam, Tanzania

## Abstract

**Background:**

Tanzania switched the antimalarial first line to sulphadoxine-pyrimethamine (SP) in 2001 from ineffective chloroquine (CQ). By 2003 higher levels of SP resistance were recorded, prompting an urgent need for replacing the first line drug with ACT, as currently recommended by the World Health Organization. Despite this recommendation country-specific evidence-based data to support efficacy and safety profile of ACT is still limited. A study on the efficacy and safety of artesunate plus amodiaquine (AS+AQ) and artemether plus lumefantrine (AL)(Coartem^®^) was carried out in 2004 with the view of supporting the National Malaria Control Programme in the review of the policy in mainland Tanzania.

**Methods:**

An *in vivo *efficacy study was conducted at Ipinda and Mlimba health facilities between May and November 2004. The study recruited children aged 6–59 months presenting with symptoms of uncomplicated malaria, history of fever or an axillary temperature ≥37.5°C; mono infection with *Pasmodium falciparum *(2,000–200,000 parasites/μl). Patients were randomized to received either SP or amodiaquine monotherapy or treated with standard doses of AS+AQ in Mlimba and Coartem in Kyela and followed-up for 28 days to assess treatment responses. This study reports results of the combination therapies.

**Results:**

A total of 157 children (76 in Mlimba and 99 in Kyela) who were enrolled in to the study and treated with either AL or AS+AQ were successfully followed-up. Both combinations were tolerated and effected rapid fever and parasite clearance. The crude ACPRs were 80 (87%) and 41 (63%) for AL and AS+AQ respectively. However, after PCR adjustments the corresponding figures raised to 100% (n = 86) and 93.8% (n = 45) in AL and AS+AQ groups, respectively. The mean haemoglobin improved moderately from day 0 to day 28 by 1 g/dl in AL and 0.4 g/dl in AS+AQ treatment group and was statistically significant (p < 0.001 both).

**Conclusion:**

These findings provide substantial evidence that AL is highly efficacious in areas of high resistance of SP and supported the country's decision to switch from SP monotherapy to AL.

## Background

The emergence and spread of *Plasmodium falciparum *resistance to commonly used antimalarials such as chloroquine (CQ) and sulphadoxine/pyrimethamine (SP) has posed major challenges to malaria control in sub-Saharan Africa. In the face of escalated resistance to these widely used and long utilized antimalarial the World Health Organization (WHO) currently recommends the use of artemisinin combination therapies (ACTs) as the first line treatment of malaria in sub-Saharan Africa [1-3]. Several countries in the region have started implementing the use of ACTs as the first line drug. Despite these recommendations, country specific evidence-based data to support antimalarial first line treatment policy change to ACTs is still limited[4].

In 2001, the Tanzanian Ministry of Health and Social Welfare switched its first line drug from CQ to SP Prior to this change, CQ has remained in use as the first line drug for over 45 years and had recorded day 14 parasitological cure rates of 10.3% [5]. A number of lessons were, therefore, learnt after this policy revision. Some of these include, poor acceptability of the new policy as health services providers and the general public at large were short of preparedness to adopt the new policy[6,7]. In addition, there were very few efficacious, safe and cheap drugs to be considered for first line. At the same time efficacy and safety data on the few available drugs were missing. In the aftermaths of interim policy inception, several major steps were taken including conducting *invivo *studies on efficacy of SP and other newly registered antimalarials geared to increase choices and preparedness should the need for policy revision arise [8-10]. In this framework, therefore, an *invivo *study was carried out on the efficacy of some ACT drugs with a view of supporting the National Malaria Control Programme (NMCP) in reviewing the antimalarial drug treatment policy in Tanzania.

## Methods

### Study site and design

The study was conducted within the *invivo *efficacy testing framework of the Tanzania NMCP/East African Network for Monitoring Antimalarial (EANMAT). Two health facilities took part in the study; Ipinda in Kyela District at the boarder with Malawi and Mlimba in Kilombero District in South-eastern Tanzania. This study was conducted in 2004 between January – June and March – October at Ipinda and Mlimba health facilities, respectively. At both sites malaria transmission is perennial and peaks between May and July, after the long seasonal rainfall.

The WHO standardized protocol for the assessment of therapeutic efficacy of antimalarial drugs (WHO 2000) was used and the study included sick children who were 6–59 months age if they presented with history of fever in the past 24 hours or axillary temperature of ≥37.5°C, and mono-infection of *P. falciparum *count of 50–5,000/200 white blood cells (WBC) assumed to be 2,000–200,000 parasites/μl. Patients were excluded from the study if they present with repeated convulsion, inability to take anything orally, severe anaemia i.e. Hb ≤ 5 gm/dl, difficult in breathing or patient with signs consistent with renal failure and patient's parent/guardian unwillingness to participate.

### Intervention

The patients were randomized to receive either monotherapy or the ACT according to the NMCP schedule of sentinel testing. At Ipinda sentinel site SP and amodiaquine were used as the monotherapies and artemether/lumefantrine (AL)(Coartem^®^) was used as the ACT while artesunate and amodiaquine (AS+AQ) in an *ad-hoc *as ACT and the same monotherapies were given at Mlimba. This study reports only the outcome of both combination therapies. AL was given to children according to bodyweight as follows 20/120 mg tablet to those weighing 5–14 kg and two tablets 40/240 mg to children with 15–24 kg. The full course of treatment for all study patients in this group consisted of 6-doses of AL that was given at 0, 8 and 16 hours, the remaining doses were given at 12-hourly intervals for a total of three days. At Mlimba sentinel site AS+AQ was given at a dose of 4 mg/kg and 10 mg/kg body weight respectively. AS+AQ was given on day 0, 1 and 2 with only amodiaquine's dose reduced to 5 mg/kg body weight on day 2. All treatments were supervised by study nurse and patients were observed for 30 minutes in the aftermath of drug intake. All patients who vomited within 30 minutes intervals were re-administered another full dose of the same medicine. All treated patients were followed for 28 days to assess clinical and parasitological responses. Patients that did not turn up for scheduled dose were visited at home by the study nurse on the same day. Treatment outcomes were classified as early treatment failure (ETF), late clinical failure (LCF), late parasitological failure (LPF) and adequate clinical and parasitological responders (ACPR) using the (WHO 2003) guidelines. Clinical therapeutic outcomes were adjusted by genotyping the *P. falciparum *merozoite surface protein 2 (*msp2*) and glutamate rich protein (*glurp*) on admission (Day 0) and any day of infection recurrences (Day 7, 14, 21 or 28). Recrudescence was differentiated from new-infections as described by Mugittu et al 2006 [11] only for patients who received ACTs due to cost limitation. Under this assessment, only parasitaemia that was confirmed by PCR as recrudescence was considered as treatment failure and conversely, was considered as new infection and counted as the ACPR.

### Clinical and laboratory procedures

Patient follow up were scheduled on days 1, 2, 3,7,14, 21 and 28. On each visit, including day 0 under which patient were enrolled. Clinical, parasitological and haematological examinations were performed. Haemoglobin level (Hb) was assessed using HemoCue^® ^(Angelholm, Sweden) and the Hb of ≤5 g/dl was considered as severe anaemia. Parasitological examinations involved preparation of thick and thin blood smears from each patient and examined by specialized microscopist from Ifakara Health Research and Development Centre (IHRDC). Parasitaemia was expressed as count per 200 WBC of blood assuming a normal leucocytes level of 8,000/μl.

### Analysis

Data generated in patient's case record forms were entered on FoxPro^® ^database software version 7 (Microsoft Corporation, Redmond USA 2001) at Ifakara Health Research and Development Centre (IHRDC). Data analysis was performed using STATA^® ^statistical analysis software package version 8 (Stata corporation, Collage Station TX, USA, 2003). Descriptive analysis was done and differences in proportions of treatment outcome were compared using chi square test for proportions. Student's t-test was applied for continuous variables. Data on patients that were excluded for different reasons and those that were loss to follow up were not considered in the final analysis.

## Results

### Study profile and patient's records

A total of 99 and 76 patients were enrolled at Ipinda and Mlimba health facilities, respectively. Table 1 summarizes patient's mean age, body weights, clinical and haematological parameters at admission/enrollments. There were 18 patients who were loss to follow up; 7/99 (7%) in Ipinda and 11/76 (14.5%) in Mlimba. Overall 92/99 (92.9%) patients in Ipinda (AL arm) and 65/76 (85.5%) in Mlimba (AS+AQ arm) were available for the assessment of therapeutic endpoints.

**Table 1:** Mean age, temperature, parasite density and haemoglobin on the day of enrollments

**Sentinel site**	**Measured parameters-Means (Standard Deviations [SD])**				
	
	*Age in years (SD)*	*Body weight in Kg (SD)*	*Temperature in °C (SD)*	*Hb in g/dl(SD)*	*Parasites/μl (SD)*
Ipinda (n = 99)	2.3 (1.2)	11.9 (2.7)	38.9 (0.98)	9.7 (1.4)	43114.5 (40130.4)
Mlimba (n = 76)	2.1 (1.2)	11.3 (2.5)	38.2 (1.2)	8.7 (1.7)	49347.8 (48194.1)

### Treatment outcome

Both drugs were tolerated; there was no report of significant Adverse Drug Reaction (ADR). Table 2 shows crude and PCR corrected treatment rates of the test drugs. The crude ACPRs were 80/92 (87%) and 41/65 (63%) for AL and AS+AQ, respectively. After PCR adjustment however, the corresponding figures rose to 100% (86/86) and 93.8% (45/48) in AL and AS+AQ respectively. Most of the recurrent infections at both sites were due to LPF. Interestingly, after genotyping all these were found to be due to new infection. The study recorded only two recrudescent infection, both of which in the AS+AQ. No recrudescence was observed in the AL arm. Moreover, a total of 10 recurrent infections (six in AL and four in AS+AQ) could not be unresolved even after repeated DNA extraction and PCR amplification. When these 10 recurrent infections were assumed to be recrudescence in the final analysis together with the PCR corrected ones however, the ACPRs were 93.3% (83/89) in AL and 87.1% (54/62) in AS+AQ.

**Table 2:** Clinical and parasitological therapeutic outcome

	**AL (Ipinda site)**			**AS+AQ (Mlimba site)**		
	
**End points**	Before PCR corrections	After PCR corrections		Before PCR corrections	After PCR corrections	
				
		With unresolved PCR	Without unresolved PCR		With unresolved PCR	Without unresolved PCR
**ETF**	0 (0.00)	0 (0.00)	0 (0.00)	1 (1.5)	1 (1.6)	1 (2.0)
**LCF**	1 (1.0)	1 (1.1)	0 (0.00)	4 (6.1)	3 (4.8)	2 (4.2)
**LPF**	11 (12.0)	5 (5.6)	0 (0.00)	19 (29.2)	4 (6.5)	0 (0.00)
**ACPR**	80 (87.0)	83 (93.3)	86 (100)	41 (63.2)	54 (87.1)	45 (93.8)

**Total**	92	89	86	65	62	48

The mean haemoglobin improved moderately from day 0 to day 28 by 1 g/dl in AL and 0.4 g/dl in AS+AQ treatment groups. The mean haemoglobin recovery after day 28 was statically significant in both groups (p < 0.001 both).

## Discussion

The main goal of this *invivo *ACT efficacy study was to support the establishment of evidence-based results that can be used to change malaria treatment policy in Tanzania. This study has demonstrated high therapeutic efficacy and tolerability for a six dose regimen of AL and a 3-day course of AS+AQ in southern Tanzania. Both crude and PCR-corrected ACPRs for AL (87% and 100%) were higher than those recorded in the AS+AQ (63.2% and 93.8%) arm. The AL crude and PCR-corrected cure rates are more or less similar to those recorded in Muheza Tanzania in 2005 (79% and 97.2%), [12] and in mult-country (Tanzania inclusive) AL efficacy study (86.5% and 93.9%) [13].

The testing of each drug independently for each site was due to the arrangements in place that required testing each individual drug as part of antimalarial nationwide drug testing allocation. This allows accumulation of evidence of the performance of the ACT in different settings. It can be argued that there are differences from place to place related to the response to treatment that is observed but treatment policies are formulated at regional or sub-regional level hence the need to get regional summary estimates. The sentinel system is a good approach toward addressing that need. Secondly it will be very difficult to sample all possible places where variation is being expected and under the current malaria transmission intensity in Tanzania, it is hard to follow up enough patients for all treatment groups in a single site during the same transmission year [14].

An interpretation of the comparative efficacy of the two drugs was not considered in our analysis. The reasons for this approach are; first this study was not designed to measure the differences between two test drugs but only to get point estimates of the efficacy which also allows monitoring of their performance overtime. Second, both ACT drugs have high efficacy profiles in the region that in principal would require huge sample size to be able to show a comparative difference [12,15]. For this same reason the direct comparison of the two ACTs in a randomized trial is not an optimal approach of monitoring drug efficacy in the programme's implementation setting and the reliance of sentinel sites in a country, therefore, becomes an efficient solution.

## Conclusion

According to the current malaria treatment policy revision guidelines (changing policy at >10% failure rate), both AL and AS+AQ would be considered suitable drugs for first line purpose since both recorded high PCR-corrected efficacies i.e. 100% and 93.8%, respectively. However, it was more rational to adopt AL as its efficacy is far above the cut-off point, whereas AS+AQ efficacy is just 3 units higher from the policy revision cut-off point and its useful therapeutic life might be compromised sooner following widespread use. In addition, the policy revision process took into account the suggestion that apart from reasonably high efficacies of partner drugs, an effective combination therapy should comprise of drugs that have not been deployed previously in the area for use as monotherapies [16]. Prior to the 2006 policy change AQ was being used as a second line antimalarial drug in Tanzania. Finally, AL was highly recommended based on its extra privileges such as being the only co-formulated ACT at the time of policy change and good effectiveness parameters in the country[17]. All these experience have paved the way for adoption of AL as the first line antimalarial drug in Tanzania.

## Authors' contributions

AM was responsible for the protocol development, study design, field trial set up data analysis and developing of the manuscript. SA was responsible for study design, training research assistants and developing the manuscript. KM participated in manuscript development and for comments on the earlier version. DS participated in molecular genotyping of recurrent infections. RM & AM were responsible for study design.

## References

[B1] WHO Antimalarial Drug Combination Therapy: Report of a WHO Technical Consultation. WHO/CDS/RBM.

[B2] 1093 , WHO/UNICEF (2003). Africa Malaria report.. MAL.

[B3] Koram KA, Abuaku B, Duah N, Quashie N (2005). Comparative efficacy of antimalarial drugs including ACTs in the treatment of uncomplicated malaria among children under 5 years in Ghana. Acta Trop.

[B4] Coleman PG, Morel C, Shillcutt S, Goodman C, Mills AJ (2004). A threshold analysis of the cost-effectiveness of artemisinin-based combination therapies in sub-saharan Africa. Am J Trop Med Hyg.

[B5] Hatz C, Abdulla S, Mull R, Schellenberg D, Gathmann I, Kibatala P, Beck HP, Tanner M, Royce C (1998). Efficacy and safety of CGP 56697 (artemether and benflumetol) compared with chloroquine to treat acute falciparum malaria in Tanzanian children aged 1-5 years. Trop Med Int Health.

[B6] Mubyazi GM, Gonzalez-Block MA (2005). Research influence on antimalarial drug policy change in Tanzania: case study of replacing chloroquine with sulfadoxine-pyrimethamine as the first-line drug. Malar J.

[B7] Eriksen J, Nsimba SE, Minzi OM, Sanga AJ, Petzold M, Gustafsson LL, Warsame MY, Tomson G (2005). Adoption of the new antimalarial drug policy in Tanzania--a cross-sectional study in the community. Trop Med Int Health.

[B8] Mugittu K, Ndejembi M, Malisa A, Lemnge M, Premji Z, Mwita A, Nkya W, Kataraihya J, Abdulla S, Beck HP, Mshinda H (2004). Therapeutic efficacy of sulfadoxine-pyrimethamine and prevalence of resistance markers in Tanzania prior to revision of malaria treatment policy: Plasmodium falciparum dihydrofolate reductase and dihydropteroate synthase mutations in monitoring in vivo resistance. Am J Trop Med Hyg.

[B9] Hetzel MW, Msechu JJ, Goodman C, Lengeler C, Obrist B, Kachur SP, Makemba A, Nathan R, Schulze A, Mshinda H (2006). Decreased availability of antimalarials in the private sector following the policy change from chloroquine to sulphadoxine-pyrimethamine in the Kilombero Valley, Tanzania. Malar J.

[B10] Tarimo DS, Minjas JN, Bygbjerg IC (2001). Perception of chloroquine efficacy and alternative treatments for uncomplicated malaria in children in a holoendemic area of Tanzania: implications for the change of treatment policy. Trop Med Int Health.

[B11] Mugittu K, Adjuik M, Snounou G, Ntoumi F, Taylor W, Mshinda H, Olliaro P, Beck HP (2006). Molecular genotyping to distinguish between recrudescents and new infections in treatment trials of Plasmodium falciparum malaria conducted in Sub-Saharan Africa: adjustment of parasitological outcomes and assessment of genotyping effectiveness. Trop Med Int Health.

[B12] Mutabingwa TK, Anthony D, Heller A, Hallett R, Ahmed J, Drakeley C, Greenwood BM, Whitty CJ (2005). Amodiaquine alone, amodiaquine+sulfadoxine-pyrimethamine, amodiaquine+artesunate, and artemether-lumefantrine for outpatient treatment of malaria in Tanzanian children: a four-arm randomised effectiveness trial. Lancet.

[B13] Falade C, Makanga M, Premji Z, Ortmann CE, Stockmeyer M, de Palacios PI (2005). Efficacy and safety of artemether-lumefantrine (Coartem) tablets (six-dose regimen) in African infants and children with acute, uncomplicated falciparum malaria. Trans R Soc Trop Med Hyg.

[B14] Schellenberg D, Menendez C, Aponte J, Guinovart C, Mshinda H, Tanner M, Alonso P (2004). The changing epidemiology of malaria in Ifakara Town, southern Tanzania. Trop Med Int Health.

[B15] Adjuik M, Agnamey P, Babiker A, Borrmann S, Brasseur P, Cisse M, Cobelens F, Diallo S, Faucher JF, Garner P, Gikunda S, Kremsner PG, Krishna S, Lell B, Loolpapit M, Matsiegui PB, Missinou MA, Mwanza J, Ntoumi F, Olliaro P, Osimbo P, Rezbach P, Some E, Taylor WR (2002). Amodiaquine-artesunate versus amodiaquine for uncomplicated Plasmodium falciparum malaria in African children: a randomised, multicentre trial. Lancet.

[B16] Watkins WM, Sibley CH, Hastings IM (2005). The search for effective and sustainable treatments for Plasmodium falciparum malaria in Africa: a model of the selection of resistance by antifolate drugs and their combinations. Am J Trop Med Hyg.

[B17] Mulligan JA, Mandike R, Palmer N, Williams H, Abdulla S, Bloland P, Mills A (2006). The costs of changing national policy: lessons from malaria treatment policy guidelines in Tanzania. Trop Med Int Health.

